# Mucosal Immunoregulatory Properties of *Tsukamurella inchonensis* to Reverse Experimental Food Allergy

**DOI:** 10.3389/fimmu.2021.641597

**Published:** 2021-04-30

**Authors:** Paola L. Smaldini, Fernando M. Trejo, Gastón P. Rizzo, Diego J. Comerci, Jaap Kampinga, Guillermo H. Docena

**Affiliations:** ^1^ Departamento de Ciencias Biológicas, Instituto de Estudios Inmunológicos y Fisiopatológicos (IIFP), UNLP, CONICET, asociado a CIC PBA, Facultad de Ciencias Exactas, La Plata, Argentina; ^2^ Instituto de Investigaciones Biotecnológicas, Dr. Rodolfo A. Ugalde (IIB-INTECH), CONICET, Universidad Nacional de San Martín, Buenos Aires, Argentina; ^3^ ActinoPharma Ltd., London, United Kingdom

**Keywords:** *Tsukamurella inchonensis*, Food hypersensitivity, Anti-inflammatory agents, Enterocytes, Intestinal mucosa

## Abstract

The intestinal mucosa is lined by epithelial cells, which are key cells to sustain gut homeostasis. Food allergy is an immune-mediated adverse reaction to food, likely due to defective regulatory circuits. *Tsukamurella inchonensis* is a non-pathogenic bacterium with immunomodulatory properties. We hypothesize that the anti-inflammatory effect of dead *T*. *inchonensis* on activated epithelial cells modulates milk allergy through the restoration of tolerance in a mouse model. Epithelial cells (Caco-2 and enterocytes from mouse gut) and macrophages were stimulated with *T*. *inchonensis* and induction of luciferase under the NF-κB promoter, ROS and cytokines production were studied. Balb/c mice were mucosally sensitized with cow´s milk proteins plus cholera toxin and orally challenged with the allergen to evidence hypersensitivity symptoms. After that, mice were orally administered with heat-killed *T*. *inchonensis* as treatment and then challenged with the allergen. The therapeutic efficacy was *in vivo* (clinical score and cutaneous test) and *in vitro* (serum specific antibodies and cytokines-ELISA, and cell analysis-flow cytometry) evaluated. Heat-killed *T*. *inchonensis* modulated the induction of pro-inflammatory chemokines, with an increase in anti-inflammatory cytokines by intestinal epithelial cells and by macrophages with decreased OX40L expression. *In vivo*, oral administration of *T*. *inchonensis* increased the frequency of lamina propria CD4^+^CD25^+^FoxP3^+^ T cells, and clinical signs were lower in *T*. *inchonensis*-treated mice compared with milk-sensitized animals. *In vivo* depletion of Tregs (anti-CD25) abrogated *T*. *inchonensis* immunomodulation. In conclusion, these bacteria suppressed the intestinal inflammatory immune response to reverse food allergy.

## Introduction

Cow’s milk allergy (CMA) is an immunological mediated reaction to cow’s milk proteins and one of the most prevalent human food allergies, particularly in infants and young children (1). Evidence of a lack of oral tolerance in food allergic patients (2, 3) has increased the interest in oral immunotherapy (OIT) as an option for disease-modifying therapy. Palforzia is the only immunotherapy approved for food allergies. It has proved to raise the threshold at which an allergic individual will react to accidental exposure to peanut (https://www.fda.gov/news-events/press-announcements/fda-approves-first-drug-treatment-peanut-allergy-children). Nevertheless, safety and efficacy are still being investigated in a wide spectrum of corrective immunotherapies, and further research is needed to identify the network of regulatory circuits that could be induced to mitigate the tissue inflammation (4, 5). Moreover, animal models have provided important information for the understanding and development of novel therapies. We and others demonstrated that the adoptive transfer of CD4^+^CD25^+^ regulatory T cells suppressed food allergy and the eosinophilia induced by the exposure to a specific antigen in the gut and airways, respectively (6, 7). It has also been described that patients with mutations in FoxP3 (Immune dysregulation, polyendocrinopathy, enteropathy syndrome or IPEX) have no regulatory T cells. Patients suffer from a generalized autoimmune enteropathy after birth, with high IgE levels and eosinophilia, accompanied by thyroiditis, type I diabetes, severe eczema, gastrointestinal disorders, etc.

Our studies demonstrated that the heat-killed *Gordonia bronchialis*, an Actinomyces bacteria, suppressed the NF-κB pathway on intestinal epithelial cells, which controlled the induction of mucosal type-2 cytokines, the secretion of IgE and the onset of hypersensitivity symptoms on sensitized mice following the oral challenge with the allergens (8). Here, we examined the immunomodulatory capacity of the heat-killed Actinomyces *Tsukamurella inchonensis* (Ti) to reverse an allergen-specific Th2 immune response when it was administered through the oral route. We observed that ROS induction promoted IL-10 secretion and IL-10^+^IFN-γ^+^ Treg expansion, which ameliorated the allergic reaction. Our findings may offer new insights on immunomodulatory properties of heat-killed *T. inchonensis* to be exploited in immunotherapeutic strategies for food allergies.

## Material and Methods

### Bacteria

Heat-killed, borate-buffered, *Tsukamurella inchonensis* was provided by Actinopharma. The samples were provided as autoclaved suspensions at a concentration of 100 mg wet mass.

*Bifidobacterium bifidum* CIDCA5310, was isolated from healthy infant feces as indicated in Gomez Zavaglia et al. (9). Bifidobacteria were grown in MRS (Man, Rogosa and Sharpe) medium (BIOKAR, Biokar Diagnostics, Beauvais, France) plus cysteine (0.05% w/v) at 37°C for 20 h under anaerobic conditions (AnaeroPak; Mitshubishi Gas Chemical Co, Inc.). The pathogenic *Clostridium difficile* strain 117 is a clinical isolate obtained at Hospital Muñiz (Buenos Aires, Argentina) (10). *Clostridium* bacteria were grown under anaerobic conditions in BHI (Brain Heart Infusion) (BIOKAR, Biokar Diagnostics, Beauvais, France) plus cysteine (0.05% w/v) at 37°C for 20 h. Plate counts were performed by plating serial dilutions of the cultures on BHI-agar or MRS-agar for *C difficile* and MRS-agar, respectively. Plates were incubated for 72 h at 37°C in anaerobic conditions. *Salmonella typhimurium* was grown in trypticase soy broth medium at 37°C overnight and diluted 1:100, and grown from early to mid-log phase. This culture was diluted in phosphate buffer saline (PBS) to an A_600_ of 0.2 and the suspensions were plated onto trypticase soy agar to calculate cell concentrations. To obtain heat-killed *Salmonella*, the culture was autoclaved.

### Cell Cultures

Human colon carcinoma cell lines Caco-2 and Caco-2-luciferase (Caco-luc) were employed. In Caco-luc cells, the luciferase expression is under the control of the CCL20 promoter, which, in turn, is controlled through different NF-κB binding sites (11). The macrophage cell line RAW 264.7 was also employed. Cells were grown in DMEM supplemented with 10% of fetal bovine serum (FBS, Gibco Thermo Fisher Scientific, Waltham, MA, USA).

Bone marrow-derived dendritic cells (BMDC) were obtained with cells removed from the femur of Balb/c mice (purchased from the School of Animal Science, University of La Plata, La Plata, Argentina) under sterile conditions. Cells were rinsed from the bone marrow with PSE buffer (PBS with 0.5% of FBS plus 2 mM of EDTA, pH 7.2), centrifuged at 500xg for 5 min and the cell pellet was resuspended with ACK (0.15M NH4Cl, 10 mM KHCO3, 0.1 mM Na2EDTA, pH 7.2) or red blood cell lysis buffer. The cell suspension was centrifuged, and the pelleted cells were washed with PBS and resuspended in 10 mL of RPMI-1640 medium supplemented with 10% FBS in a culture dish at a density of 1×10^6^ cell/mL. Thereafter, GM-CSF (Peprotech, Princeton, NJ, USA) was added into the medium to a final concentration of 20 ng/mL and cells were cultured for 3 days. Fresh medium supplemented with GM-CSF was added, and on day 7 the culture medium was replaced. On day 8, the semi-suspended cells and loosely attached cells were collected by gently pipetting the medium against the plate. The cells were plated into 48-well plates and exposed to bacteria or immunomodulatory agents.

Intestinal epithelial cells (IEC) were isolated from adult Balb/c mice and cultured in a collagen membrane as described in Di Claudio et al. (12). Briefly, animals were sacrificed, and the first 5 cm of the jejunum were removed under sterile conditions and washed with PBS supplemented with antibiotics (penicillin 100 U/mL and streptomycin 100 μg/mL) and 20% FBS for 10 min at room temperature. The gut was opened longitudinally, and mucus was removed by incubation with medium and 1 mM dithiothreitol for 10 min at 4°C. After that, the tissue was incubated with medium and 0.5 mM EDTA with orbital agitation for 30 min at 37°C to remove the epithelial compartment (the remaining tissue was discarded). Cells were washed, then plated in complete medium on collagen membranes for 8 days and incubated at 37°C in a humidified atmosphere containing 5% CO2 and then, they were exposed to bacteria or immunomodulatory agents.

### Reporter Gene Expression Assay

Caco-luc cells were incubated with flagellin (FliC, 1ug/mL), heat-killed or live *S. typhimurium* (ratio 1:100) or with *Tsukamurella inchonensis* (ratio 1:100). To evaluate Ti modulation, cells were pre-incubated with the bacteria for 30 min before FliC was added. Cells were incubated for 6 h at 37°C in a humidified atmosphere containing 5% CO2. Then cells were washed with PBS and lysed with lysis buffer (25 mM Tris-phosphate, 2mM DTT, 2mM 1,2-diaminocyclohexane-N,N,N0,N0-tetraacetic acid, 10% glycerol and 1% Triton X-100) and luciferase activity was measured using the luciferase substrate (luciferase assay kit; Promega, Madison, WI, USA) following the manufacturer’s instructions. Light emission was measured with a luminometer (Luminoscan TL Plus; Labsystem, Jerusalem, Israel). The relative luciferase activity was determined as the ratio of treated/untreated cell activities, and FliC was used as a control of 100% induction of luciferase expression.

### Reactive Oxygen Species Assessment

For intracellular ROS production, cells were loaded with 10 μM of H_2_DCFDA (Sigma, St Louis, Mo, USA). Cells were pretreated with diphenyleneiodonium 5μM (DPI), an NAD(P)H oxidase inhibitor, or with the Indoleamine-2,3-dioxydase (IDO) inhibitor 1-methyl-tryptophan (MT) 500 μM for 30 min. Then cells were exposed to Ti *(*ratio 1:100*)*, or H_2_O_2_ 1 mM as a positive control. After aspirating the culture medium and washing the cells, freshly H_2_DCFDA in DMEM was added and incubated for 30 min at 37°C in a humidified atmosphere (5% CO_2_).

Macrophages were detached from the plate by adding cell dissociation buffer, enzyme-free PBS (Gibco, Thermofisher, San Diego, CA, USA), washed with HEPES-buffered saline solution (HBSS) and centrifuged (500 xg). Pelleted cells were resuspended and analyzed by flow cytometry using FACS Calibur cytometer (Becton Dickinson, New Jersey, USA).

After the incubation, Caco-2 cells were washed and HEPES-buffered saline solution (HBSS) was added and ROS production was analyzed by immunofluorescence using a Nikon Eclipse Ti-U microscope (Nikon, Tokyo, Japan).

### Kynurenine Measurement

To monitor IDO enzyme activity, macrophages were incubated in a medium containing 100 μM tryptophan (Life Technologies, Grand Island, NY) and incubated for 24 hours with Ti (ratio 1:100), IFN-γ (100 ng/mL) or H_2_O_2_ (1 mM), as inhibitor MT (500 μM) or DPI (5 μM) were pre-incubated for 30 min. Supernatants were harvested and assayed for the presence of kynurenine by a spectrophotometric assay. Briefly, fifty microliters of 30% trichloroacetic acid were added to 100 μL culture supernatant, vortexed, and centrifuged at 8000x*g* for 5 minutes. The Ehrlich reagent (100 mg *P*-dimethyl benzaldehyde, 5 mL glacial acetic acid) (75 μL) was added to 75 μL of supernatants and immediately the optical density (OD) was measured at 492 nm in a microplate reader (Sirio S SAECS, BIOARS, Buenos Aires, Argentina).

### Quantification of Secreted Cytokines

Intestinal epithelial cells, RAW cells or BMDC were plated in complete medium and exposed to Ti (ratio 1:100), *Clostridium difficile* (ratio 1:100) or *Bifidobacterium bifidum* (ratio 1:100) at 37°C for 24 h. Supernatants were collected and the concentration of homeostatic (IL-25 and TSLP) and regulatory cytokines (IL-10 and TGF-β) were assessed by ELISA. Commercial kits were employed (IL-10, IL-25 and TGF-β- eBioscience, San Diego, CA, USA; TSLP-Biolegend, San Diego, CA, USA) according to manufacturer´s specifications. As controls, cells were pre-treated with DPI 5 μM or MT 500 μM) for 30 min.

### OX40L Expression on BMDC

BMDC were incubated with cholera toxin (CT) (Sigma, St Louis, Mo, USA) (1 μg/mL), *T*. *inchonensis* (1:100), medium as control or pre-incubated with *T*. *inchonensis* and then stimulated with CT. For flow cytometry analysis, cells were washed with PBS containing 0.5% of FBS and stained with specified PE-CD11c, PercPCy5.5-CD11b and APC-Ox40L conjugated monoclonal antibodies for 30 min (Thermofisher, San Diego, CA, USA). Stained cells were washed and analyzed by FACS Calibur. The gating strategy for the cell analysis consisted of a gate based on SSC-H *vs* FSC-H parameters, followed by CD11c *vs* OX40L fluorescence on the dot plot or OX40L^+^ cells gated on CD11c^+^ cells were quantified on a histogram.

### *In Vivo* Inhibitory Effect of *T. inchonensis* in the Gut

Balb/c mice were gavaged with a suspension of heat-killed *T. inchonensis* (10^8^ bacteria/200 ul) in saline solution on days 0 and 7, followed by 10 ug of CT per day for 3 days. Two days later, mice were sacrificed, and the gut was removed. The jejunum was washed with saline buffer, Peyer’s patches were dissected off and intestinal epithelial cells were isolated as previously described (7). Total RNA was isolated using the Illustra RNAspin mini isolation kit according to the manufacturer’s specifications (GE Healthcare, Freiburg, Germany). A cDNA preparation was obtained from 1 μg of RNA using Moloney Murine Leukemia Virus (M-MLV) reverse transcriptase and random primers (Thermofisher, San Diego, CA, USA) and *ccl20* gene expression was determined by real-time quantitative PCR using SYBRGreen fluorescence (Thermofisher, San Diego, CA, USA). *β-actin* was used as a housekeeping gene to standardize the total amount of cDNA. The fold change was defined as the ratio of normalized values corresponding to IECs of CT, Ti or CT+Ti treated mice to that treated with saline (PBS).

### *In Vivo* Assessment of Regulatory T Cells

Balb/c mice received a suspension of *T*. *inchonensis* (10^8^ bacteria in 200 ul) by gavage every day for 7 days, followed by 4 oral administration of 10ug CT to induce gut inflammation. As controls, mice received only CT, *T*. *inchonensis* or PBS. On days 21 or 28, mice were sacrificed, and Tregs were evaluated in lamina propria by flow cytometry. Moreover, spleen cells were cultured with *T*. *inchonensis* (ratio 1:100) or RPMI, and IL-10 were determined in the supernatant as previously described.

### Oral Immunomodulation With Heat-Killed *T. inchonensis* in a Cow’s Milk Food Allergy Mouse Model to Detect Treg Cells

Balb/c were sensitized with 6 weekly intragastric doses of 20 mg of cow’s milk protein (CMP) with 10 ug CT (Sensitized mice); as a control, another group of mice received only CMP (sham control). Ten days after the final boost, mice were intragastrically (i.g.) challenged with 20 mg of CMP. Simultaneously, mice received *T. inchonensis* (10^8^ bacteria/200 ul) by gavage once a week for 7 weeks (Ti+sens group), whereas control mice received PBS (Sens group). Concomitantly, mice were injected with anti-CD25 (100 μg/mouse) (PC61.5 eBioscience San Diego, CA, USA) or the isotype control antibody to deplete Treg or the isotype control (eBioscience San Diego, CA, USA).

### Reversion of the Allergic Reaction Through the Oral Administration of Heat-Killed *Tsukamurella inchonensis*

Balb/c mice were sensitized with 6 weekly intragastric doses of 20 mg of cow’s milk protein (CMP) plus 10 ug cholera toxin (Sigma). Ten days after the final boost, mice were i.g. challenged twice on the following days with 20 mg of CMP. Thereafter, mice received *T. inchonensis* (10^8^ bacteria/200 ul) by gavage once a week for 7 weeks (Ti+sens group). Oral challenges with 20 mg of milk proteins were carried out on days 40 and 74 to score hypersensitivity symptoms, and finally, two oral challenges on consecutive days were performed at days 91 and 92. Twenty-four hours following the oral challenges, animals were sacrificed. The spleen, gut and blood samples were collected.

### *In Vivo* Evaluation of the Allergic State

*-Assessment of clinical signs*. Symptoms were observed between 30 and 60 min after the oral challenge in a blinded fashion by 2 independent investigators. Clinical scores were assigned according to the following range: 0 = no symptoms; 1= scratching and rubbing around the nose and head; 2 =puffiness around the eyes and mouth, diarrhea, pilar erecti, reduced activity, and/or decreased activity with increase respiratory rate; 3 = wheezing, labored respiration, cyanosis around the mouth and the tail; 4 = no activity after prodding, or tremor and convulsion; and 5 = death.

*-Cutaneous test*. Mice were injected with 20 μg of CMP in 20 μL of sterile saline in one footpad and saline in the contralateral footpad (negative control). Then, animals were injected intravenously (i.v.) with 100 μL of 0.1% Evans blue dye (Anedra, Buenos Aires, Argentina). The blue color in the skin 30 min following the iv injection was considered a positive test. The footpad thickness was measured with a digital caliper.

### *In Vitro* Evaluation of Allergic Disease

*-Serum CMP-specific IgE, IgG1 and IgG2a detection*. For evaluating of specific IgE antibodies, serum samples were tested by EAST and for IgG isotypes, serum CMP-specific IgG1 and IgG2a antibodies were measured by ELISA as previously described (7).

*-Cytokine response of splenocytes stimulated with CMP*. Spleen cells were mechanically removed from the spleen and cultured at a concentration of 4 x10^6^ cells/well for 72 h at 37°C in the presence of RPMI or with 0.35 mg/mL CMP. Supernatants were harvested and assayed for IL-5, IL-4, IFN-γ, and IL-10 concentration by flow cytometry using the CBA commercial kit (eBioscience, San Diego, CA, USA).

*-Flow cytometry.* Intestinal lamina propria (LP) cells were isolated from the jejunum as described in Smaldini et al. (7). Briefly, the intestinal tissue was removed and digested with 400 U/mL Collagenase Type IV (Sigma, St Louis, Mo, USA) for 45 minutes at 37°C. Cell suspensions were filtered and washed in PBS solution. The cells were incubated with anti-CD4 (PerCyP 5.5) and anti-CD25 (PE) (eBioscience, San Diego, CA, USA) for membrane staining for 40 min at 4°C. For intracytoplasmatic staining, cells were washed, pre-incubated with the fixation/permeabilization solution (eBioscience, San Diego, CA, USA) for 20 min at 4°C, and then incubated with the Staining Intracellular kit (eBioscience, San Diego, CA, USA) with anti-FoxP3 (APC) (eBioscience, San Diego, CA, USA). Cells were analyzed with the FACS Calibur cytometer (BD, NJ, USA) using QuestProCell software. The gating strategy for the cell analysis consisted of a lymphocyte gate based on SSC-H *vs* FSC-H parameters, followed by SSC-H *vs* CD4 fluorescence. Then, the CD4^+^ lymphocyte subset was gated as CD25 *vs* FoxP3 or CD4^+^FoxP3^+^ followed by IFN-γ *vs* IL-10. Finally, data were analyzed with the FlowJo software.

### Statistical Analysis

All statistical analysis was carried out using GraphPad Prism 8 software. The significance of the difference was determined using one or two-way ANOVA followed by Bonferroni’s test and Student´s *t-*test; p-value <0.05 was considered as statistically significant.

Cultures assays were done 3 times. Each dot plot represents the median of triplicates of each experiment. Animal experiments were done 2 times; each dot plot represents a mouse of one representative experiment.

## Results

### Heat-Killed *Tsukamurella inchonensis* Promoted Immunomodulation of Epithelial Cells

**Figure 1:**
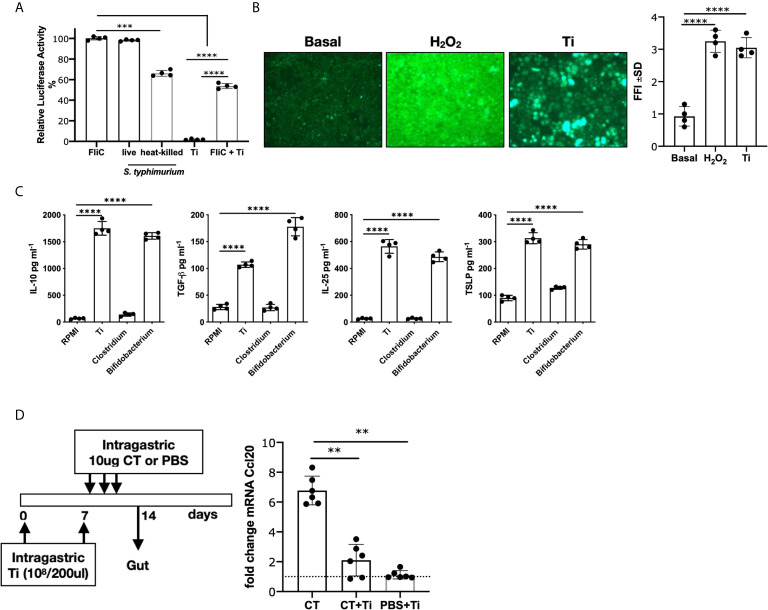
Effect of *Tsukamurella inchonensis* on epithelial cells. **(A)** Caco-luc cells were incubated with 1ug/mL flagellin (FliC), heat-killed or live *Salmonella typhimurium* or with Ti (ratio 1:100). Cells were pre-incubated with Ti for 30 min and then exposed to FliC for 6 h and light emission was measured. **(B)** Induction of ROS by Ti. ROS were revealed by fluorescence microscopy with H_2_DCFDA on Caco-2 cells incubated with Hepes-HBSS, Ti (ratio 1:100) and 1mM H_2_O_2_. **(C)** Mouse primary intestinal epithelial cells were cultured for 8 days and then stimulated with Ti*, Bifidobacterium bifidum* CIDCA5310 and *Clostridium difficile* 117 for 24 h (ratio 1:100). Quantification of cytokines in supernatants were analyzed by ELISA. **(D)** *In vivo* modulation of Ccl20 mRNA expression. Balb/c mice (n=6 in duplicate) were treated with Ti (10^8^/200 μL) and the gavage with 3 doses of CT (10μg/dose). Ccl20 mRNA expression was analyzed on enterocytes by RT-qPCR. Dots in culture assays represent a mean of triplicate values. Statistics significant difference with ANOVA test: ****p<0.0001, ***p<0.001, **p<0.01.

We first examined the effect of Ti on intestinal epithelial cells, and we observed that heat-killed Ti did not activate the reporter Caco-2 luciferase cell line, whereas it significantly suppressed the FliC-induced NF-κB-dependent cell activation. As controls, we tested *Salmonella typhimurium*, live or heat-killed, which induced cell activation (**Figure 1A**). In agreement with our previous results in which another Actinomyces induced the production of ROS and a regulatory effect (8), we exposed Caco-2 cells to Ti or peroxide as control, and we found that Ti promoted ROS production (p<0.0001) (**Figure 1B**). We then assessed the production of regulatory cytokines on a primary mouse intestinal epithelial cells, and we found that IL-10 and TGF-β were secreted (p<0.0001). We also found an increased secretion of IL-25 and TSLP, homeostatic intestinal cytokines, by Ti-exposed cells (p<0.0001). As controls, cells were incubated with the pathogenic *Clostridium difficile* and the probiotic *Bifidobacterium bifidum* (**Figure 1C**), showing that the latter had similar behavior to Ti, whereas the former did not induce these inhibitory cytokines. To further evaluate Ti’s *in vivo* immunomodulatory effect of Ti, mice were i.g. administered with the heat-killed bacteria and then the pro-inflammatory cholera toxin adjuvant. We observed that Ti promoted a significant reduction of the CT-driven intestinal inflammation (**Figure 1D**) and the local tissue expression of *ccl20*, a chemokine that is produced by intestinal epithelial cells and attracts dendritic cells and T cells to initiate inflammation. The administration of Ti did not induce the ccl20 expression.

### IL-10 Was Critical for the Immunomodulatory Effect Exerted by Heat-Killed *Tsukamurella inchonensis*

**Figure 2:**
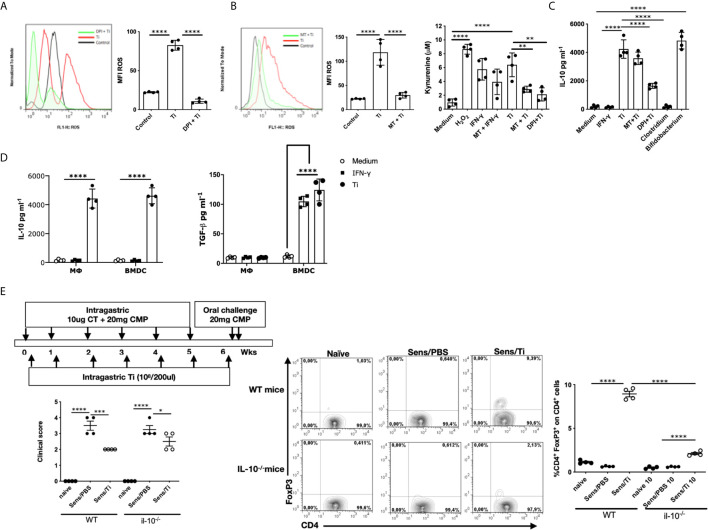
Importance of IL-10 for the immunomodulatory response. **(A)** Induction of ROS by Ti (ratio 1:100) on macrophages, and 5 μM of diphenylene iodonium (DPI) was used as NDPH inhibitor. **(B)** Induction of ROS and kynurenine quantification in the presence of Ti (ratio 1:100), 100 ng/mL IFN-γ was used as an IDO inducer, 5μM DPI or 500μM 1-Methyltryptophan (MT) were used as NADPH or IDO inhibitors, respectively. **(C)** Quantification of IL-10 in the supernatants of macrophages by ELISA. **(D)** Quantification of IL-10 and TGF-β in the supernatants of macrophages and BMDC by ELISA. **(E)** Balb/c and Balb/c IL-10-null mice (n=4/group) were sensitized, as shown in the schematic drawing of the experimental protocol. Clinical scores were recorded 45 minutes after oral challenge with CMP. Representative staining of CD4^+^FoxP3^+^ cells gated on CD4^+^ lymphocytes cells in the jejunum lamina propria by flow cytometry. Frequencies of cells are expressed as the mean values ± SEM. Statistic significant difference with ANOVA test: ****p<0.0001, ***p<0.001, *p<0.05, **p<0.01. Cultures assays were done 4 times. Each dot plot represents the median of triplicates of each experiment. Animal experiments were done twice and each dot plot represents a mouse of one representative experiment.

Considering that antigen-presenting cells are involved in T cell activation, we next investigated Ti´s effect on macrophages and bone marrow-derived dendritic cells. First, we focused on macrophages and we exposed the murine alveolar RAW 264.7 cell line to Ti. We found that macrophages produced ROS (p<0.0001), which was significantly inhibited with the NADPH oxidase inhibitor (DPI) (**Figure 2A**). We then evaluated ROS production in the presence of Ti or Ti along with an IDO inhibitor, methyl tryptophan or MT (**Figure 2B**), and we found that the Ti induced ROS production was suppressed with MT (p<0.0001), suggesting that IDO is involved in this mechanism. To confirm this finding, kynurenine was analyzed, and we found that Ti promoted this tryptophan metabolite production. As controls, we observed that IFN-γ enhanced IDO´s action, which was reversed with the incubation of cells with MT. Moreover, Ti induced kynurenine production (p<0.0001), which was significantly suppressed with MT or DPI. To gain further insight into ROS- and IDO-dependent IL-10-production, we evaluated this cytokine’s secretion in the presence of inhibitors. We firstly found that murine macrophages produced IL-10 when exposed to Ti (p<0.0001) (**Figure 2C**). Interestingly, the inhibition of IL-10-secretion achieved with Ti and DPI indicated that ROS production was directly involved in this mechanism (p<0.0001). Conversely, we observed that IL-10 secretion was IDO-independent (**Figure 2C**). As controls, neither IFN-γ nor *Clostridium* up-regulated the IL-10 secretion; conversely, we found that *Bifidobacterium*, significantly induced IL-10 production. **Figure 2D** depicts that RAW cells cultured with Ti produced IL-10 and no TGF- β, whereas BMDC secreted IL-10 and TGF-β (p<0.0001).

To investigate the role of this regulatory circuit triggered by Ti in the induction of hypersensitivity symptoms following sensitization, wild-type and IL-10-null mice were exposed to intragastric CT/CMP and Ti, according to the schematic protocol depicted in **Figure 2E**. Despite the Treg (CD4^+^FoxP3^+^ cells) induction on lamina propria of Ti-treated sensitized wild-type mice and the significant reduction of Treg´s frequency in sensitized/treated IL-10-null mice, no statistical difference in the clinical scores were observed between sensitized mice treated with Ti in IL-10-producing or IL-10 knockout animals. We observed a reduced frequency of CD4^+^FoxP3^+^ cells and high clinical scores in sensitized wild-type and IL-10-null mice. The co-administration of Ti with cholera toxin and the allergen in IL-10 KO mice promoted a significant suppression of symptoms compared to sensitized mice, suggesting that the immunomodulatory effect exerted by Ti has somehow an IL-10-independent mechanism. Sensitized IL-10-null mice that were co-administered with Ti showed a significantly increased frequency of intestinal CD4^+^FoxP3^+^ cells, probably due to the local effect produced by TGF-β secreted by dendritic cells on Ti -specific T cells.

### Oral Administration of Heat-Killed *T. inchonensis* Induced Regulatory T Cells and IL-10 Secretion

**Figure 3:**
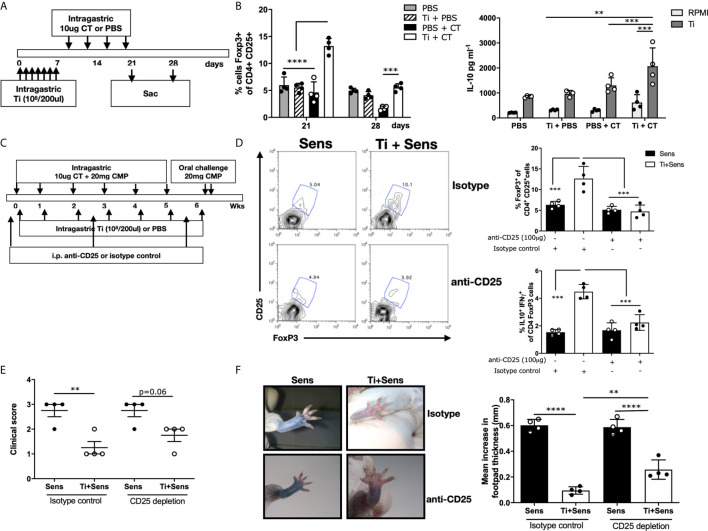
*Tsukamurella inchonensis* induces regulatory T cells *in vivo.* **(A)** The schematic protocol of sensitization and Ti treatment of Balb/c mice. **(B)** Frequency of CD4^+^CD25^+^FoxP3^+^ cells in jejunum lamina propria at days 21 and 28. Quantification of IL-10 in the supernatants of splenocytes stimulated with Ti by ELISA. **(C)** The schematic protocol of simultaneous treatment of Ti during allergic sensitization. Anti-CD25 was injected in mice (100 μg/mouse) to deplete CD25-expressing cells. **(D)** Representative staining of CD25^+^FoxP3^+^ cells gated on CD4^+^ lymphocytes in the lamina propria by flow cytometry. Analysis of CD4^+^CD25^+^ FoxP3^+^ and IL-10^+^IFN-γ of CD4^+^FoxP3^+^ cells in the lamina propria. **(E)** Clinical score corresponding to symptoms elicited following the oral challenges with milk proteins (n=4/group). **(F)** Skin tests and footpad swelling, data are expressed as the mean values ± SEM. Statistics significant difference with ANOVA test: ****p<0.0001, ***p<0.001, **p<0.01. Animal experiments were done twice, and each dot plot represents a mouse of one representative experiment.

To address if intragastric Ti promoted the Treg´s expansion, we daily administered mice for one week with heat-killed bacteria, and then the animals were treated with CT. One group of mice was sacrificed at day 21, while the other, at day 28 (**Figure 3A**). We observed a significant increase of CD4^+^CD25^+^FoxP3^+^ cells on the gut lamina propria of mice treated and then sensitized at day 21, and a lesser higher frequency, although statistically significant, at day 28 compared to sensitized animals that received PBS. Induction of systemic IL-10 production accompanied this increased cell frequency at day 28 (p<0.001) (**Figure 3B**). Mice that received Ti or CT did not show a significantly increased frequency of CD4^+^CD25^+^FoxP3^+^ cells and IL-10, suggesting that inflammation is necessary for Ti to promote the up-regulation of Treg and IL-10.

Based on previous studies in which we demonstrated that CD4^+^CD25^+^FoxP3^+^ cell transfer suppressed the allergic sensitization (7), and confirmed that IL-10 was critical in this mechanism, we co-administered Ti and CT+CMP to naïve mice that were intraperitoneally injected with anti-CD25 or the isotype control (**Figure 3C**). We observed that mice injected with anti-CD25 exhibited significantly lower Treg frequency upon the treatment with CT/CMP and Ti compared to mice that received the isotype control (4.73±0.77 *vs.* 12.66±2.93% CD4^+^CD25^+^FoxP3^+^ cells, respectively). Animals injected with the isotype control that received Ti and CT+CMP exhibited a significantly increased Treg frequency in lamina propria compared to mice that received CT+CMP. Importantly, IL10^+^IFNγ^+^CD4^+^FoxP3^+^ cells were increased in sensitized and Ti-treated animals that received the isotype control, compared to mice in which Treg cells were depleted (**Figure 3D**). However, differences in the clinical scores induced in sensitized/treated animals injected with anti-CD25 or the isotype control did not reach statistical significance. Mice depleted of Treg did not significantly increase the intensity of symptoms following the oral challenge with the allergen (**Figure 3E**). However, clinical scores were not significantly different between sensitized and sensitized/treated animals (p=0.06). Finally, skin tests showed a less intense dye leakage in animals that were depleted of Treg compared to sensitized mice (p<0.001) (**Figure 3F**). Sensitized/treated mice injected with anti-CD25 had a significantly augmented footpad swelling than mice injected with the isotype control.

### The Oral Administration of Heat-Killed *T. inchonensis* Ameliorated the Allergic Response

**Figure 4:**
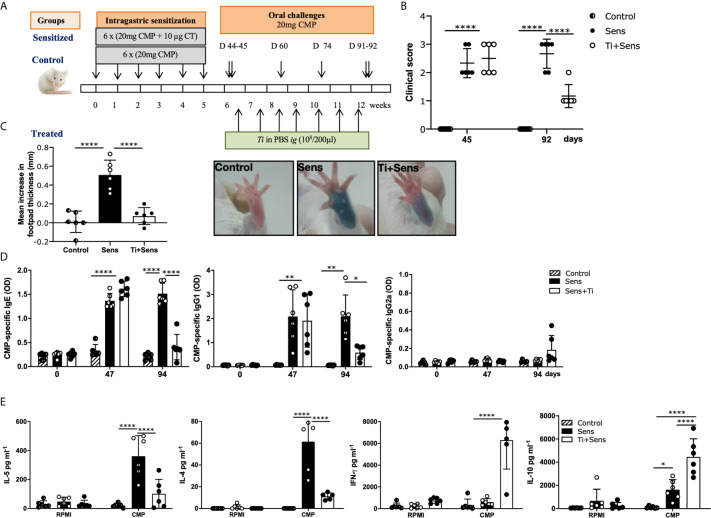
*Tsukamurella inchonensis* reverses the allergic sensitization in mice. **(A)** Balb/c mice (n=6/group) were sensitized and treated with Ti as shown in the schematic drawing of the experimental protocol. Control groups (CMP or Control and CT+CMP or Sensitized) are included. **(B)** Clinical scores of mice 30–60 min following the oral challenge with CMP; each dot represents an individual mouse. **(C)** Skin tests and footpad swelling, data are expressed as the mean values ± SEM. **(D)** Serum level of CMP-specific IgE, IgG1 and IgG2a during sensitization (day 47) and treatment with Ti (day 94). **(E)** IL-5, IL-4, IFN-γ and IL-10 concentrations in supernatants of spleen cells collected 24h following the oral challenge, which were activated with 350mg/mL CMP or RPMI, as control, for 72h. The results correspond to a single experiment representative of two separate experiments that showed similar results. Data are expressed as the mean values ± SEM. Statistic significant difference with ANOVA test: ****p<0.0001, *p<0.05, **p<0.01. Animal experiments were done twice CMP, cow’s milk proteins.

These findings prompted us to investigate if Ti could reverse intestinal inflammation and the allergic response. To address this point, sensitized mice were intragastrically treated with heat-killed Ti (Ti-treated group) or PBS (sensitized group) once a week for seven weeks in two courses; a control group received only CMP (control) as shown in the schematic protocol depicted in the **Figure 4A**. Mice were orally challenged with CMP (2 challenges) following the sensitization and the treatment steps to evidence the induction of hypersensitivity reactions minutes after the allergen exposure. **Figure 4B** shows that all mice exhibited a high clinical score after the allergic sensitization (day 45). Then, mice from the Ti-treated group showed a significant decrease in the intensity of symptoms compared to the sham group of mice (day 92). To confirm this *in vivo* suppressive effect, we carried out skin tests and we observed a reduction of footpad swelling (p<0.0001) and dye leakage in the Ti-treated mice, compared to control and sensitized mice (**Figure 4C**).

To investigate the mechanisms underlying the clinical immunomodulatory effect, serum immunoglobulins and systemic cytokines were assessed. Our findings showed that upon a rising of CMP-specific IgE during the sensitization step in all mice (p<0.0001), antibodies remained high in the sensitized group of mice at day 94. Nevertheless, animals of the Ti-treated group exhibited decreased levels of IgE compared to the sensitized group (p<0.01) (**Figure 4D**). Regarding IgG1, it was elevated in all animals at day 47, while at day 94, only Ti-treated mice showed a significant reduction of specific IgG1 (p< 0.05). We found that IgG2a remained unchanged in all animals. These results correlated significantly with the reduced secretion of the type 2 cytokines IL-4 and IL-5 by CMP-stimulated spleen cells in Ti-treated mice (p<0.0001). IL-4 and IL-5 were increased in sensitized mice (p<0.0001), whereas IFN-γ and IL-10 were significantly increased in Ti-treated animals (**Figure 4E**).

**Figure 5:**
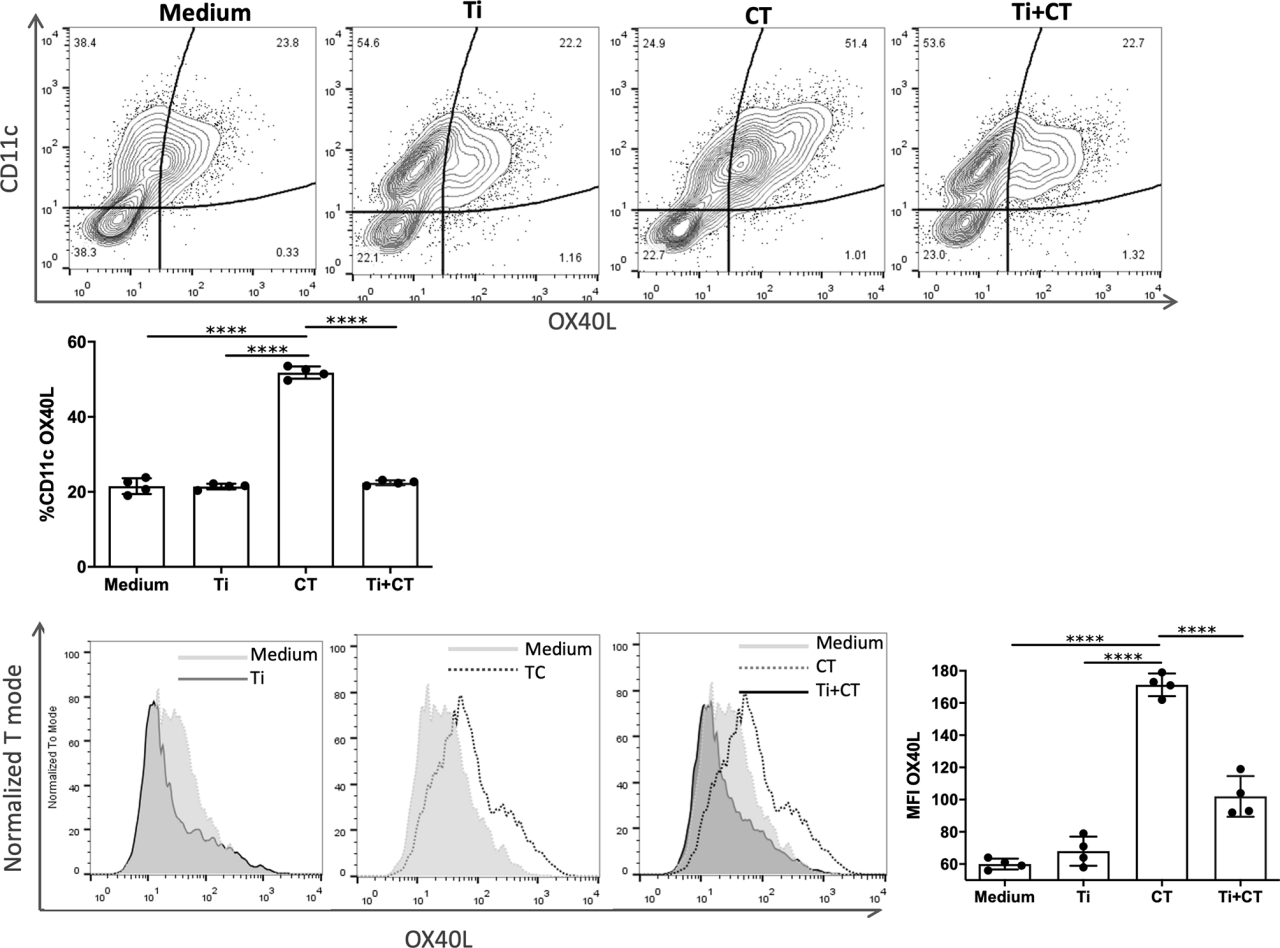
*Tsukamurella inchonensis* inhibits the expression of OX40L on bone marrow derived-dendritic cells. **(A)** BMDC from Balb/c mice were pre-incubated Ti and then stimulated with 10ug/mL CT. The membrane expression of OX40L was assessed by flow cytometry. The experiment was done in triplicate and the histogram depicted corresponds to a representative result. Data are expressed as the mean values ± SEM. Statistic significant difference with ANOVA test: ****p<0.0001.

We finally sought to elucidate the mechanisms by which the Th2-mediated reactions were controlled. The OX40/OX40L interaction contributes to an optimal T cell response following allergic stimuli and it plays an important role in the maintenance and reactivation of memory T effector cells (13). For this reason, we investigated the role of Ti on OX40L-induction on dendritic cells. Bone marrow-derived dendritic cells stimulated with CT showed an increased cell membrane expression of OX40L (p<0.0001). The co-incubation of BMDC with CT and Ti showed a significant reduction of OX40L expression (**Figure 5**). Furthermore, Ti did not induce the expression of OX40L. These findings suggest that Ti may control the induction of Th2 cells by modulating this costimulatory molecule on dendritic cells.

## Discussion

Heat-killed Actinomyces, such as *Mycobacterium obuense* or *Mycobacterium vaccae*, have shown to be safe and promising as immunotherapeutic agents in different clinical trials, which resulted in a significant improvement in the clinical outcome in patients with different cancers (14). Their action may be based on the adjuvant effect described on innate cells, thus enhancing immune response or immunomodulation (15–17). Our work highlights a previous study in which the heat-killed *Gordonia bronchialis*, an actinomycetal bacteria, prevented the activation of NF-κB on intestinal epithelial cells (8). Here, we used heat-killed Ti, another Actinomyces, to immunomodulate the allergic condition established in a pre-clinical IgE-dependent mouse model of food allergy. We first showed that heat-killed Ti induced peroxide production on intestinal epithelial cells with the secretion of IL-10, TGF-β, IL-25 and TSLP. *T. inchonensis* also promoted ROS production and suppressive cytokine secretion on macrophages and dendritic cells. A similar effect has been observed for lung dendritic cells that have been exposed to TSLP (18). Remarkably, heat-killed Ti inhibited intestinal epithelial cells’ activation *in vitro* (with flagellin) and *in vivo* (with cholera toxin), thus demonstrating its immunomodulatory effect.

Although ROS are generally considered pro-inflammatory for protection against bacterial infection, it has also been involved in regulatory circuits to maintain homeostasis and resolve infectious and noninfectious inflammation. Reduced production of IL-10 and a hyperinflammatory phenotype were reported in mice with a deficiency of ROS production (14) and in patients lacking NADPH oxidase (19). Mechanistically, IDO function and the kynurenine regulatory pathway have been reported to be dependent on ROS production (20) to control the inflammasome pathway (21). In this work, the non-pathogenic and heat-killed Ti promoted ROS production in intestinal epithelial cells and macrophages, which was inhibited with diphenylene iodonium along with the IL-10 secretion by macrophages. Interestingly, our findings showed that Ti induced ROS, IDO activation with kynurenine production, and IL-10 secretion. Nevertheless, heat-killed Ti seemed to promote ROS through a direct mechanism and an IDO-dependent pathway, whereas IL-10 secretion depended only on ROS production. The inhibition of IDO did not interfere with the production of this cytokine but with ROS production. We observed that IFN-γ induced IDO activity with increased kynurenine levels, and this metabolite promoted ROS production, which exerted the IL-10 secretion. These findings suggest that the IDO catalytic activity is directly involved in enhancing ROS production, although it is not required for IL-10 secretion. The action of the IFN-γ-inducible IDO might be more related to an immunoregulatory effect of the TGF-β axis. It has been described in fibroblasts that IFN-γ and TGF-β interplay during inflammation to restore homeostasis, suggesting that intestinal homeostasis is under tight immunological control. According to our findings, BMDC produced TGF-β upon stimulation with IFN-γ, which might be related to the IFN-γ-induced expression of IDO (22). Although further studies are warranted to ascertain whether Ti may enhance the axis ROS-IDO-TGF-β to restore homeostasis through the action of IFN-γ, our data demonstrated that in the absence of IL-10, a complementary regulatory circuit mitigated the hypersensitivity symptoms triggered with the pro-Th2 cholera toxin. According to results obtained in the IL-10 knockout mice, the induction of intestinal CD4^+^FoxP3^+^ cells was highly dependent on the presence of IL-10. Furthermore, IL-10 production and CD4^+^CD25^+^FoxP3^+^ T cells were highly dependent on the CT-driven inflammatory process in the gut. A robust and potential argument to understand this point may be that the IL-2 produced by milk-specific T cells activated in the presence of cholera toxin and milk proteins provided the IL-2 necessary for the expansion of Treg, which do not produce this T cell growth factor. However, Treg´s depletion did not completely abrogate the immunomodulation achieved with Ti in the presence of IL-10-producing Treg. The nexus between the control of allergic symptoms and Th2-mediated immunity induced with Ti and the complementary role of IL-10 and TGF- β perhaps deserves increased attention to be further investigated.

Different microorganisms or derived metabolites have been studied to control the allergic reaction (23–25) and the underlying immunological mechanisms. Here, we presented data on the mucosal administration of a non-pathogenic bacteria to reverse an established Th2-mediated allergic response with the control of IgE and IgG1 secretion, the suppression of IL-4 and IL-5 production, the inhibition of skin test and the allergic symptoms upon exposure to the offending allergen. Mechanisms by which homeostasis was restored through the oral administration of heat-killed Ti were probably achieved by the intestinal induction of IL-10, TGF-β and IFN-γ, which promoted the allergen-specific expansion of Treg and other immunomodulatory cells. Similar results were observed by Zuany-Amorim et al. that treated mice with killed *Mycobacteriun vaccae* suspension and found an increased frequency of regulatory T cells, which conferred protection against airway inflammation. This protection was mediated by IL-10 and TGF-β (26).

To gain further insight into the mechanism that may be involved in the control of the Th2-mediated immune response, we analyzed the expression of OX40L on dendritic cells as a critical co-stimulatory signal to generate Th2 cells (27–29). Our data showed that Ti abrogated the CT-driven up-regulation of OX40L on BMDC. Consequently, *T. inchonensis* may exert two potential mechanisms on innate cells to control the immune response: the induction of different regulatory circuits capable of controlling innate and adaptive immune cells and shaping dendritic cells for a type-2 immune response induction. For the latter, further investigation is worth pursuing.

In conclusion, our data show the anti-inflammatory properties of heat-killed *T. inchonensis* that exerts a wide modulation of inflammatory mechanisms. The enhanced secretion of IL-10 by intestinal epithelial cells, macrophages, dendritic cells and T cells from inflamed intestinal tissue, coordinately with TGF-β, restored mucosal homeostasis and efficiently limited excessive immune responses against dietary antigens in an allergic setting. The oral administration of the non-pathogenic and heat-killed *T. inchonensis* may have potential implications for food allergy immunotherapeutic approaches.

## Data Availability Statement

The raw data supporting the conclusions of this article will be made available by the authors, without undue reservation.

## Ethics Statement

The animal study was reviewed and approved by Comite Institucional de Cuidado y Uso de Animales de Laboratorio CICUAL, Facultad de Ciencias Exactas de la Universidad Nacional de La Plata.

## Author Contributions

Conception and design of study: GHD, JK, and PLS. Acquisition of data: PLS, FMT, and GPR. Analysis and/or interpretation of data: PLS, FMT, GPR, DJC, JK, and GHD. Manuscript preparation: PLS and GHD. Critical revision: PLS, FMT, GPR, DJC, JK, and GHD. All authors contributed to the article and approved the submitted version.

## Funding

This work was supported by the Agencia Nacional de Promoción Científica y Tecnológica (grant PICT 2015-1648) and the Consejo Nacional de Investigaciones Científicas y Técnicas (grant PIP 2013-189) to GD.

## Conflict of Interest

Author JK was employed by ActinoPharma Ltd.

The remaining authors declare that the research was conducted in the absence of any commercial or financial relationships that could be construed as a potential conflict of interest.
